# Relation between quantitative coronary CTA and myocardial ischemia by adenosine stress myocardial CT perfusion

**DOI:** 10.1007/s12350-016-0393-7

**Published:** 2016-02-09

**Authors:** Alexander R. van Rosendael, Lucia J. Kroft, Alexander Broersen, Jouke Dijkstra, Inge J. van den Hoogen, Erik W. van Zwet, Jeroen J. Bax, Michiel A. de Graaf, Arthur J. Scholte

**Affiliations:** 1 Department of Cardiology, Heart and Lung Center, Leiden University Medical Center, Albinusdreef 2, 2333 ZA, 2300 RC Leiden, The Netherlands; 2 The Interuniversity Cardiology Institute of the Netherlands, Utrecht, The Netherlands; 3 Department of Radiology, Leiden University Medical Center, Leiden, The Netherlands; 4 Division of Image Processing, Department of Radiology, Leiden University Medical Center, Leiden, The Netherlands; 5 Department of Medical Statistics and Bio-informatics, Leiden University Medical Center, Leiden, The Netherlands

**Keywords:** Coronary artery disease, quantitative coronary CTA, myocardial CT perfusion, imaging, myocardial ischemia

## Abstract

**Background:**

Coronary-computed tomography angiography (CTA) has limited accuracy to predict myocardial ischemia. Besides luminal area stenosis, other coronary plaque morphology and composition parameters may help to assess ischemia. With the integration of coronary CTA and adenosine stress CT myocardial perfusion (CTP), reliable information regarding coronary anatomy and function can be derived in one procedure. This analysis aimed to investigate the association between coronary stenosis severity, plaque composition and morphology and the presence of ischemia measured with adenosine stress myocardial CTP.

**Methods and Results:**

84 patients (age, 62 ± 10 years; 48% men) who underwent sequential coronary CTA and adenosine stress myocardial CT perfusion were analyzed. Automated quantification was performed in all coronary lesions (quantitative CTA). Downstream myocardial ischemia was assessed by visual analysis of the rest and stress CTP images and defined as a summed difference score of ≥1. One or more coronary plaques were present in 146 coronary arteries of which 31 (21%) were ischemia-related. Of the lesions with a stenosis percentage <50%, 50%-70%, and >70%, respectively, 9% (6/67), 18% (9/51), and 57% (16/28) demonstrated downstream ischemia. Furthermore, mean plaque burden, plaque volume, lesion length, maximal plaque thickness, and dense calcium volume were significantly higher in ischemia-related lesions, but only stenosis severity (%) (OR 1.06; 95% CI 1.02-1.10; *P* = .006) and lesion length (mm) (OR 1.26; 95% CI 1.02-1.55; *P* = .029) were independent correlates.

**Conclusions:**

Increasing stenosis percentage by quantitative CTA is positively correlated to myocardial ischemia measured with adenosine stress myocardial CTP. However, stenosis percentage remains a moderate determinant. Lumen area stenosis and lesion length were independently associated with ischemia, adjusted for coronary plaque volume, mean plaque burden, maximal lesion thickness, and dense calcium volume.

## Introduction

Coronary-computed tomography angiography (CTA) is an established technique to detect or rule out coronary artery disease (CAD) and correlates well with invasive coronary angiography (ICA).^1^,^2^ However, obstructive CAD (≥50% luminal narrowing) detected by coronary CTA has limited value to predict myocardial ischemia.^3^ Moreover, hemodynamically significant stenosis in patients with stable CAD is important for prognosis and the need for revascularization.^4^ Previous studies demonstrated that besides luminal area stenosis, additional plaque characteristics are related to myocardial ischemia.^5^,^6^ Quantification of stenosis severity, plaque composition and morphology can nowadays be performed with dedicated post-processing data software (quantitative CTA).^7^ Until recently, the hemodynamic consequence of an obstructive lesion detected by coronary CTA was obtained by additional nuclear or echocardiographic ischemia testing or invasive fractional flow reserve (FFR) measurement. A relatively new technique, which can be performed in addition to coronary CTA in the same setting, is adenosine stress myocardial CT perfusion (CTP) which provides functional information of a coronary stenosis.^8^

The relationship between luminal narrowing, additional coronary plaque characteristics measured with quantitative CTA, and myocardial ischemia detected by adenosine stress myocardial CTP is unknown. Accordingly, the present study aimed to explore the association of quantitatively assessed coronary plaque characteristics with myocardial ischemia by adenosine stress CTP.

## Methods

### Patients

The population consisted of 115 consecutive patients with new onset chest pain who were referred from the outpatient clinic from March 2013 till December 2014 for cardiac CT. All patients underwent coronary CTA and subsequently adenosine stress CTP at the same day. Until March 2014, all referred patients underwent both scans. After March 2014, directly after the acquisition of the coronary CTA, the presence of obstructive CAD (≥50% stenosis) was assessed on-site by an experienced physician. Only in case of obstructive CAD, additional adenosine stress CTP was performed. Patients with previous myocardial infarction, percutaneous coronary intervention, coronary artery bypass graft, anomalous coronary arteries, or insufficient image quality in one or both CT scans were excluded. Contraindications were atrial fibrillation, renal insufficiency, second or third degree atrio-ventricular block, known allergy to iodine-containing contrast agents and pregnancy. Clinical data were prospectively entered into the departmental electronic information system (EPD-Vision©, Leiden University Medical Center, The Netherlands) and retrospectively analyzed. According to the Dutch law, no Institutional Review Board approval is required for this retrospective analysis of clinically acquired data.

### Cardiac CT Acquisition

Sequential coronary CTA and adenosine stress myocardial CTP were both performed using a 320 detector row volumetric scanner (Aquilion ONE, Toshiba Medical Systems, Otawara, Japan). The effective radiation exposure was calculated by multiplying the dose-length-product by 0.014 mSv·mGy^−1^·cm^−1^.^9^

### Coronary CTA Acquisition

Patients with a heart rate exceeding 60 beats·minute^−1^ received 25-150 mg of oral metoprolol 1 hour before the scan, unless contraindicated. If the heart rate remained above 60 beats·minute^−1^, up to 15 mg of intravenous metoprolol was administrated additionally. First, a low-dose non-contrast enhanced scan was performed to determine the coronary artery calcium score and to assess the needed coverage (120-160 mm) depending on the craniocaudal length of the heart. The coronary CTA was performed with a peak tube voltage between 100 and 135 kV and tube current between 140 and 580 mA, depending on body habitus. The detector collimation was 320 × 0.5 mm, gantry rotation time was 350 ms, and temporal resolution was 175 ms. The contrast agent (Iomeron 400, Bracco, Milan, Italy) was injected in a triphasic contrast injection protocol: first, 50-90 mL (depending on patient weight) contrast agent (flow rate 5-6 mL·s^−1^), followed by 20 mL of a 1:1 mixture of contrast and saline and finally 25 mL of saline (flow rate 3 mL·s^−1^). Amounts and injection protocol were equal for coronary CTA and adenosine stress CTP and varied between 60 and 90 mL based on patient weight. Prospective ECG triggering was used to cover 70%-80% of the R-R interval. In patients with a heart rate >65 beats·minute^−1^ or irregular heart rate, 30%-80% of the R-R interval was covered. Real-time bolus tracking was performed in the descending aorta, with a threshold of 300 Hounsfield Units (HU). Reconstructed left ventricle data acquired from the coronary CTA scan served as the rest myocardial perfusion study.

### Adenosine Stress Myocardial CTP Acquisition

At least 20 minutes after finishing the coronary CTA scan, adenosine (0.14 mg·kg^−1^·minute^−1^) was administered intravenously during continuous ECG monitoring. After 4 minutes of adenosine infusion, contrast agent was administrated. Once the target threshold of 300 HU was reached in the descending aorta, adenosine stress myocardial CTP images were acquired the next one to two heartbeats depending on patient heart rate, covering 80%-99% of the R-R interval. The tube settings were the same as for the coronary CTA.

### Quantitative CTA

Quantitative CTA was performed to provide a detailed and objective assessment of the coronary plaque. Dedicated software (QAngio CT Research Edition; Medis Medical Imaging Systems, Leiden, The Netherlands) was used to perform the automated quantitative analysis. As previously described, the software automatically identifies the vessels and detects the contours of the vessel wall and the lumen.^5^,^7^ If needed, the observer could manually adjust these contours. Automated quantification for each coronary lesion in all coronary arteries was performed. Reference lines for the lumen and the outer vessel wall were created using non-bifurcated, non-diseased segments proximal and distal to the lesion. Subsequently, a reference slope for lumen and outer vessel wall was created between these reference segments, which served as a reference frame of a non-diseased artery. This reference slope represented the normal proximal to distal tapering of a coronary artery. Quantitative CTA parameters were automatically generated by using the plaque contours in relation to the reference slope. In the absence of coronary plaques, no analysis was performed and these arteries were excluded from further analysis. Percentage lumen area stenosis, lumen diameter stenosis, mean plaque burden, plaque volume, lesion length, maximal plaque thickness, fibrous volume, fibro-fatty volume, necrotic core volume, dense calcium volume, and remodeling index were derived from each coronary plaque. Plaque constitution (fibrous, fibro-fatty, necrotic core, and dense calcium) was automatically assessed using adaptive HU thresholds: meaning that HU thresholds for plaque composition are adapted according to lumen contrast attenuation variations, as demonstrated previously.^10^ Definitions of the parameters are presented in Table 1.

**Table 1:** Definitions of quantitative CTA-derived parameters

Parameter	Definition
Lumen area stenosis (%)	Percentage of lumen area stenosis at level of MLA. 1 − (MLA/corresponding reference lumen area) × 100%
Lumen diameter stenosis (%)	Percentage diameter stenosis at the MLA
Mean plaque burden (%)	Sum of ((vessel wall area − lumen area)/vessel wall area) per slice/number of slices
Lesion length (mm)	Distance between proximal and distal ends of the plaque
Maximal plaque thickness (mm)	Maximal distance between vessel wall and lumen
Remodeling index	Vessel wall area/corresponding reference vessel wall area at the level of the MLA

CTA, computed tomography angiography; MLA, minimal lumen area

The most severe lesion in each coronary artery was selected by maximum lumen area stenosis percentage. Coronary arteries were defined as left anterior descending artery (LAD), right coronary artery (RCA), and left circumflex artery (LCX). Plaques in diagonal branches were allocated to the LAD, and plaques in the intermediate branch were allocated to the LCX. Plaque characteristics were compared between ischemia-related and non-ischemic coronary lesions. To clarify a possible incremental relation with myocardial ischemia, lumen area stenosis was divided into three groups (<50%, 50%-70%, and >70%).^11^

### Adenosine Stress and Rest Myocardial CTP Analysis

All myocardial CTP images were analyzed and interpreted by two trained observers with dedicated post-processing software (Vitrea FX 6.5; Vital Images, Minnetonka, Minnesota, USA). Coronary CTA and myocardial perfusion analyses were done independently from each other to reduce bias caused by knowledge of the other scan result. Data were arranged in the short axis, vertical long axis, and horizontal long axis with a slice thickness of 3 mm. Data from the coronary CTA were used for the rest myocardial CTP. The phase with the best image quality was selected and interpreted with a narrow window width and level setting (W300/L150), according to the 17-myocardial segment model.^12^ The best phase was defined as the phase with the least artifacts. The observers were allowed to adjust the display settings after the initial exploratory reading. Each segment was scored for the presence of perfusion defects. Inter-observer variability was dissolved by consensus.

**Figure 1:**
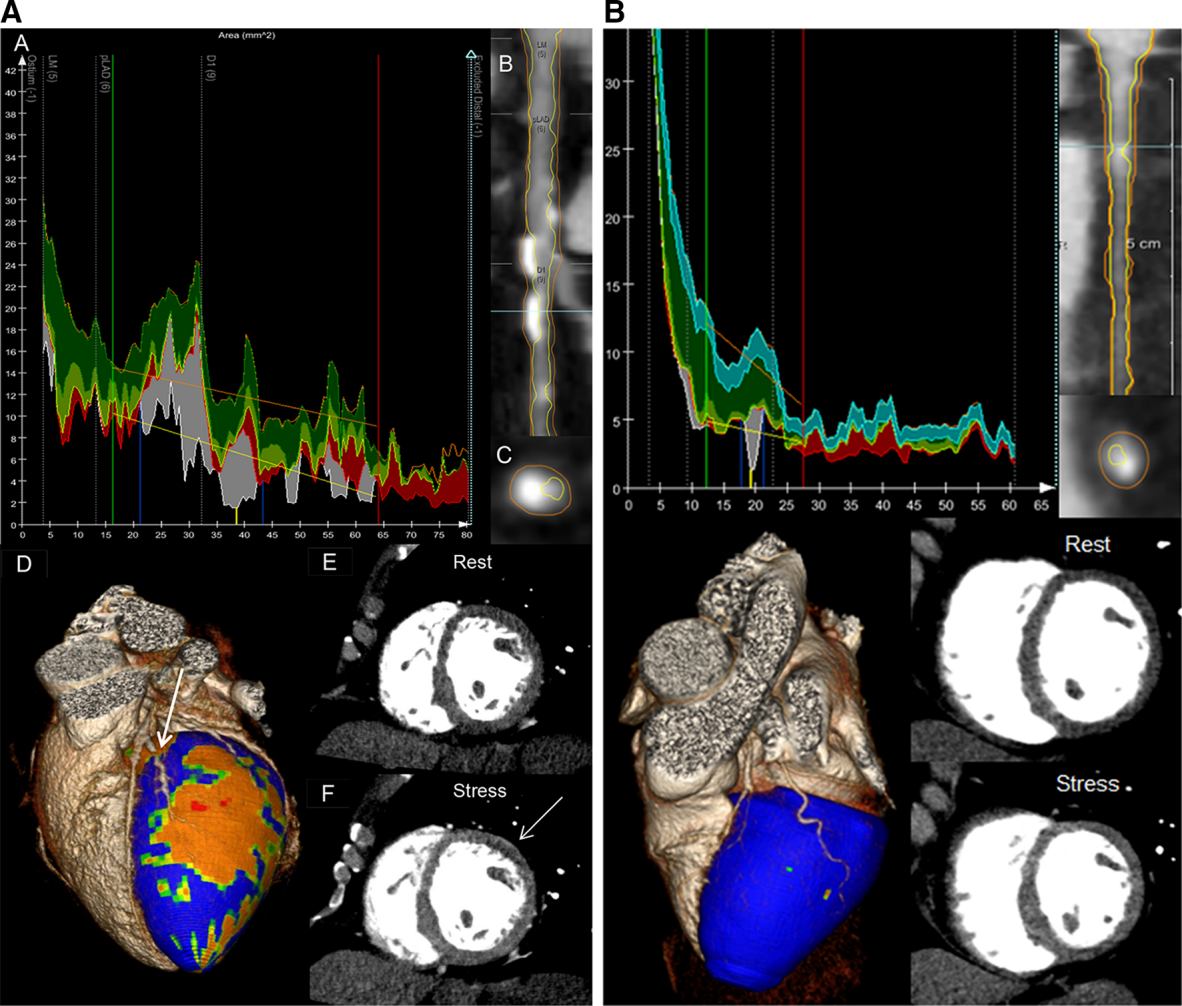
(**A**) Quantitative CTA and adenosine stress CTP of an ischemic lesion. Example of a 58-year-old female patient with a lesion in the first diagonal branch and corresponding myocardial ischemia. *A* Automated quantitative CTA of the artery was performed. The *blue lines* were set proximal and distal to the lesion. The *green* and *red lines* represent non-diseased coronary artery segments proximal and distal to the lesion. The *yellow* and *orange lines* represent the reference markers for, respectively, the lumen and vessel wall. The *vertical yellow line* is placed at the site of maximal stenosis percentage: 76.6%. Furthermore, mean plaque burden was 79.9%; plaque volume: 129.3 mm^3^; lesion length: 19.0 mm; maximal plaque thickness: 2.6 mm; dense calcium volume: 36.6 mm^3^. *B* Longitudinal lumen and vessel wall contours. *C* Transverse lumen and vessel wall contours at the site of maximal stenosis percentage. *D* 3D fusion of the coronary CTA and myocardial hypo-perfusion during adenosine stress (*orange*, *red*). A stenosis in the first diagonal branch (*arrow*) is depicted with corresponding myocardial ischemia. *E* Rest CTP study showing normal myocardial enhancement. *F* Adenosine stress myocardial CTP showing a small anterolateral subendocardial perfusion defect (*arrow*). (**B**) Quantitative CTA and adenosine stress CTP of a non-ischemic lesion. Same data reconstructions as shown in (**A**). A non-ischemic coronary lesion in the proximal LCX is depicted. Maximal stenosis percentage was: 69.1%. Mean plaque burden was: 67.2%; plaque volume: 27.1 mm^3^; lesion length: 3.5 mm; maximal plaque thickness: 1.8 mm; dense calcium volume: 9.4 mm^3^. Despite the high stenosis percentage, lesion length, maximal plaque thickness, and dense calcium volume were relatively low, resulting in normal myocardial enhancement on adenosine stress

Fixed defects were defined as the persistence of a perfusion defect in the same myocardial segment at rest and during adenosine stress myocardial CTP. In case of an abnormal scan, all phases (both systolic and diastolic) were examined to better differentiate between potential artifacts and real perfusion defects. The persistence of a hypo-enhanced area in multiple phases suggests true perfusion defect, as a motion artifact usually does not persist in multiple phases.^13^ To assess the hemodynamic significance of each coronary lesion, corresponding myocardial ischemia was defined as a summed difference score by adenosine stress CTP ≥ 1. Each myocardial segment was matched to its corresponding epicardial coronary artery, using a standard model.^12^ The anteroseptal and anterior segments were matched to the LAD, the lateral to the LCX, and the inferoseptal and inferior segments to the RCA. If a mismatch existed while using the standard coronary artery-myocardial segment alignment and multiple arteries were supplying the ischemic myocardium based on vessel tracking, the artery with obstructive CAD was related to the perfusion defect. Three-dimensional fusion of coronary CTA and adenosine stress myocardial CTP images by Vitrea software was used to facilitate the linking of regional myocardial ischemia with its corresponding coronary artery, as depicted in Figure 1.

### Statistical Analysis

Continuous data were presented as mean ± standard deviation. When non-normally distributed, data were presented as medians with 25th and 75th percentiles. Categorical data were presented as frequencies and percentages and were compared with the Chi square test. Quantitative CTA parameters of lesions with and without downstream myocardial ischemia were analyzed with the independent-samples *t* test or the Mann-Whitney test, as appropriate. Multivariate logistic regression analysis was performed with the plaque characteristics that were significantly different between ischemic and non-ischemic lesions; ischemia was the dependent variable. Values were expressed as odds ratios (OR) with 95% confidence interval (CI). A two-sided *P* value less than .05 was considered statistically significant. All statistical analyses were performed with the use of IBM SPSS Statistics software (version 20, IBM Corp, Armonk, New York, USA).

## Results

### Patients

A total of 115 patients underwent both coronary CTA and adenosine stress myocardial CTP. Excluded were 31 patients, because of insufficient image quality (n = 12), previous PCI (n = 8) or CABG (n = 3), anomalous coronary arteries (n = 6), and the presence of a fixed perfusion defect (indicating prior myocardial infarction) by myocardial CTP (n = 2). The 84 remaining patients were included in the present analysis. Clinical characteristics are presented in Table 2. In total, 40 (48%) patients were men and the mean age was 62 (±10) years. The prevalence of risk factors for coronary artery disease was high: 32% had diabetes, 58% hypertension, 44% hypercholesterolemia, 13% were currently smoking, and 50% had a family history of CAD. The median calcium score was 98 (IQR 19-330). Effective radiation exposure for the coronary CTA was 2.6 mSv (IQR: 1.7-3.7) and for adenosine stress CTP: 3.2 mSv (IQR: 2.3-4.6). The dose for both scans was 6.4 mSv (IQR: 4.3-8.6).

**Table 2:** Clinical characteristics

Baseline characteristics (n = 84)	Values
Men	40 (48)
Age (years)	62 ± 10
Calcium score	98 (19–330)
Diabetes	27 (32)
Hypertension*	49 (58)
Hypercholesterolemia^†^	37 (44)
Current smoking	11 (13)
Positive family history^‡^	42 (50)
Beta-blocker	40 (48)
ACE-I/ARB	39 (46)
Calcium antagonist	16 (19)
Statin	38 (45)
Acetyl salicylic acid	25 (30)

Data are represented as mean ± standard deviation, median (IQR), or as number (percentage).

*ACE*-*I*, Angiotensin converting enzyme inhibitor; *ARB*, angiotensin receptor blocker.

* Systolic blood pressure ≥140 mm Hg, diastolic blood pressure ≥90 mm Hg, or use of antihypertensive mediation.

^†^ Serum total cholesterol ≥230 mg·dL^−1^ or serum triglycerides ≥200 mg·dL^−1^ or treatment with lipid-lowering drugs.

^‡^ Presence of CAD in 1st degree family members at <55 years in men and <65 years in women.

### Quantitative CTA

Of the 252 coronary arteries in total, at least one coronary plaque (calcified, mixed or non-calcified) was present in 146 (58%) arteries. 67 (42%) plaques showed a stenosis <50%; 51 (35%) plaques between 50 and 70% and 28 (19%) plaques >70% stenosis. 106 arteries did not show any coronary lesion. Downstream those, 2 perfusion defects were seen.

### Adenosine Stress Myocardial CTP

In total, 31 (21%) of the 146 coronary lesions were related to corresponding myocardial ischemia by adenosine stress CTP. The remaining 115 (79%) lesions did not demonstrate downstream ischemia. Compared with non-ischemic lesions, hemodynamic significant lesions showed a significantly higher percentage lumen area stenosis (69.0% ± 16.8 vs 49.6% ± 17.2, *P* < .001), lumen diameter stenosis (46.7% ± 16.8 vs 30.2% ± 13.1, *P* < .001), mean plaque burden (59.6% ± 8.7 vs 52.3% ± 9.9, *P* < .001), plaque volume (68.7 mm^3^ [40.2-126.0] vs 44.7 mm^3^ [24.1-80.3], *P* = .021), lesion length (12.3 mm [4.9-14.9], vs 6.5 [4.0-10.4], *P* = .033), maximal plaque thickness (2.3 mm [1.8-2.7] vs 1.9 [1.5-2.4], *P* = .021), and dense calcium volume (25.8 [7.6-39.4] vs 7.8 [0.0-20.2], *P* = .005), as presented in Table 3.

**Table 3:** Quantitative CTA parameters for ischemic and non-ischemic lesions

Quantitative CTA parameters^a^	Ischemia (n = 31)	No ischemia (n = 115)	*P* value
Lumen area stenosis (%)	69.0 ± 16.8	49.6 ± 17.2	<.001
Lumen diameter stenosis (%)	46.7 ± 16.8	30.2 ± 13.1	<.001
Mean plaque burden (%)	59.6 ± 8.7	52.3 ± 9.9	<.001
Plaque volume (mm^3^)	68.7 (40.2–126.0)	44.7 (24.1–80.0)	.021
Lesion length (mm)	12.3 (4.9–14.9)	6.5 (4.0–10.4)	.033
Maximal plaque thickness (mm)	2.3 (1.8–2.7)	1.9 (1.5–2.4)	.021
Fibrous volume (mm^3^)	24.7 (14.2–43.4)	16.9 (9.6–32.9)	.068
Fibro–fatty volume (mm^3^)	3.9 (1.7–8.3)	3.6 (1.5–9.9)	.928
Necrotic core volume (mm^3^)	0.6 (0.1–2.1)	0.9 (0.1–1.9)	.661
Dense calcium volume (mm^3^)	25.8 (7.6–39.4)	7.8 (0.0–20.2)	.005
Remodeling index	1.0 (0.9–1.1)	1.0 (0.9–1.1)	.820

*CTA*, Computed tomography angiography.

^a^Results from the most severe lesion per coronary artery.

In multivariate analysis, lumen area stenosis (%) (OR 1.06; 95% CI 1.02-1.10; *P* = .006) and lesion length (mm) (OR 1.26; 95% CI 1.02-1.55; *P* = .029) were independently correlated to ischemia, when entered in the model with maximal plaque thickness (mm) (OR 1.61; 95% CI 0.50-5.18; *P* = .427), dense calcium volume (mm^3^) (OR 0.99; 95% CI 0.95-1.04; *P* = .804), mean plaque burden (%) (OR 1.04; 95% CI 0.96-1.13; *P* = .325), and plaque volume (mm^3^) (OR 0.98; 95% CI 0.96-1.00; *P* = .089). An example of a patient with a severely stenotic lesion plus high values of mean plaque burden, plaque volume, lesion length, maximal plaque thickness, and dense calcium volume that were related to ischemia is shown in Figure 1A. Figure 1B demonstrates a lesion with high stenosis percentage but relatively low plaque volume, maximal plaque thickness, lesion length, and dense calcium volume that did not cause ischemia.

### Association Between Stenosis Severity and Adenosine Stress Myocardial CTP

**Figure 2:**
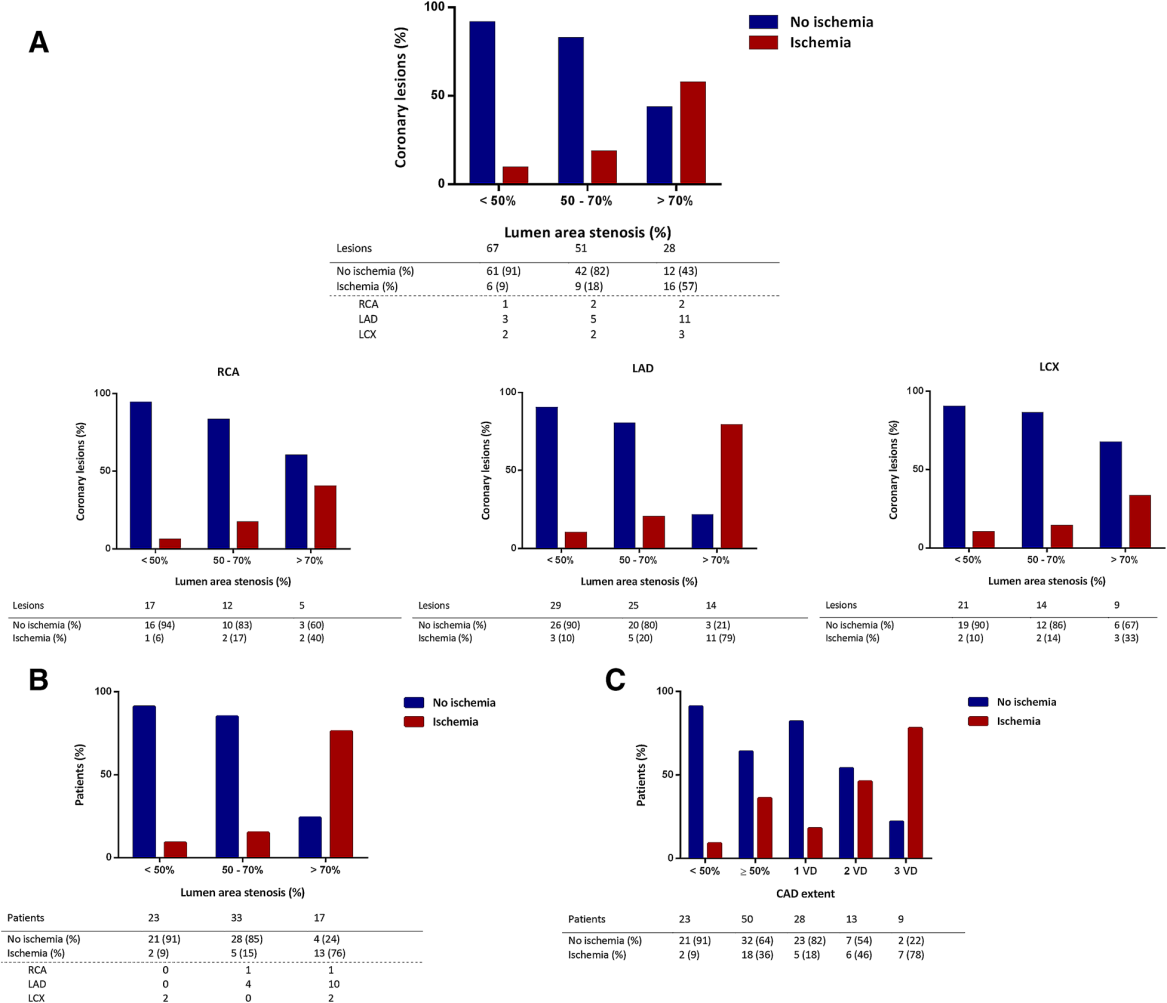
(**A**) Vessel-based analysis relating stenosis percentage to myocardial ischemia. (**B**) Patient-based analysis relating stenosis percentage to myocardial ischemia. (**C**) Extent of CAD related to myocardial ischemia. *CAD*, Coronary artery disease; *VD*, vessel with ≥50% stenosis

9% (6/67) of the coronary lesions <50% stenosis were related to myocardial ischemia. In the categories, 50%-70% and >70%, respectively, 18% (9/51) and 57% (16/28) were ischemic, as depicted in Figure 2A. Of 12 of the non-ischemic lesion >70% stenosis, only 2 were located in proximal coronary parts and 6 were located distally in side branches (intermediate, anterolateral, obtuse marginal, or diagonal branches) which may have had influence on the stenosis-ischemia relationship. In a per vessel analysis, a worse correlation between stenosis severity and ischemia was seen in the lesions >70% for RCA and LCX in comparison with the LAD. A patient-based analysis is shown in Figure 2B, revealing that if a patient had a coronary lesion >70%, 76% (13/17) of them showed myocardial ischemia. Figure 2C shows the correlation for increasing extent of CAD and ischemia: <50%, 9% (2/23); 1 vessel with ≥50% stenosis, 18% (5/28); 2 vessels with ≥50% stenosis, 46% (6/13) and 3 vessels with ≥50% stenosis, 78% (7/9).

## Discussion

The main findings of the present analysis are as follows: increasing stenosis percentage measured with quantitative CTA relates to an increasing percentage of myocardial ischemia measured with adenosine stress myocardial CTP. Second, the current quantitative CTA analysis demonstrated that hemodynamically significant lesions comprised a higher plaque and dense calcium volume, mean plaque burden, larger maximal plaque thickness, and longer lesion length, but only stenosis severity and lesion length were independent determinants.

### Stenosis Severity Vs Myocardial Ischemia

The relation between stenosis severity and myocardial ischemia has been investigated previously.^11^,^14^,^15^ Uren et al related the percentage stenosis of a coronary lesion to downstream myocardial blood flow measured with positron emission tomography (PET) and demonstrated that basal myocardial blood flow during rest remained constant regardless of stenosis severity.^11^ Moreover, only in lesions with ≥40% stenosis, myocardial flow progressively decreased during hyperaemia, indicating that lesions below 40% stenosis have no hemodynamic consequences. More studies confirmed this inverse relation between hyperaemic blood flow and stenosis severity and quantified the exact percentage of stenosis to the presence of myocardial ischemia.^16^,^17^ Di Carli et al demonstrated a significantly lower flow reserve (as measured with PET) in lesions with stenosis between 70% and 90% as compared to 50% and 70%.^16^ While stenosis severity had only a moderate impact on downstream hyperaemic myocardial flow, no difference in flow reserve was observed in lesions of 50%-70% compared with lesions below 50%.

More recently, studies using invasive FFR for assessment of hemodynamic consequences of a stenosis, confirmed that moderate lesions do not often result in myocardial ischemia. Tonino et al demonstrated with quantitative coronary angiography that lesions with a stenosis degree of 50%-70%, 71%-90%, and 91%-99%, respectively, 35%, 80%, and 96% showed a FFR ≤ 0.80.^18^ In accordance with ICA, coronary CTA is of limited value in predicting the hemodynamic significance of a stenotic lesion. Schuijf et al reported that in 114 patients who underwent coronary CTA and single-photon emission-computed tomography (SPECT) myocardial perfusion imaging, only 50% of obstructive coronary lesions were ischemia-related.^3^ Similar findings were reported by Sato et al who established that for 105 coronary lesions with 60%-70%, 70%-80%, and ≥80% stenosis, the prevalence of ischemia was, respectively, 33%, 54%, and 86%.^19^ Thus, whether assessed by ICA or coronary CTA, an intermediate to severe stenosis degree of a coronary lesion provides insufficient information for clinical decision making and requires further ischemia testing. Therefore, integration of coronary CTA with the assessment of myocardial ischemia in one session would be ideal, which has become feasible with adenosine stress myocardial CTP. George et al demonstrated the feasibility of adenosine stress myocardial CTP to detect myocardial ischemia.^20^ In a sub-study of the CORE320 trial in which 381 patients underwent adenosine myocardial stress CTP and SPECT myocardial perfusion imaging, CTP yielded a higher sensitivity (88% [CI 83-92] to 62 [CI 56-69]), but a lower specificity (55% [CI 46-63] to 67 [CI 59-75] to predict obstructive CAD (≥50% stenosis) measured with ICA.^21^

Hence, with the integrated assessment of coronary CTA and adenosine stress myocardial CTP, reliable information concerning stenosis severity and corresponding myocardial perfusion (and ischemia) can be obtained in one session. In the current study, we related quantitatively assessed coronary stenosis severity to myocardial ischemia assessed by adenosine stress myocardial CTP. In accordance to previously mentioned results, we demonstrated a moderate association between intermediate and severely stenotic lesions and myocardial ischemia, especially for the RCA and LCX. The detection of myocardial ischemia by stress myocardial CTP related to coronary anatomy has not been investigated previously. These findings may encourage to perform stress myocardial CTP after coronary CTA throughout a wide range of stenosis degree on coronary CTA.

### Adenosine Stress Myocardial CTP Vs Coronary CTA Plaque Composition

An explanation of these findings could be that obstructive CAD is just one manifestation of atherosclerosis. Other mechanisms as coronary vasospasm, inflammation, microvascular dysfunction, endothelial dysfunction, and thrombosis relate to myocardial ischemia as well.^22^ Besides stenosis severity, plaque composition and morphology may also contribute to the development of ischemia. Indeed, recent studies demonstrated that certain plaque characteristics were independently associated with the presence of ischemia.^6^,^23^,^24^

First, Park et al reported the incremental value of spotty calcifications, low attenuation plaque, positive remodeling, and percent aggregate plaque volume (%APV) to predict ischemia.^6^ %APV represents the total plaque volume as function of total vessel volume from the ostium till the distal part of the lesion, indicating the patient’s total atherosclerotic burden. Nakazato et al confirmed the independent and incremental value of %APV for ischemia (assessed by FFR).^23^

Second, Naya et al reported that ischemia measured with PET correlated with the modified Duke CAD index (which indicates the total atherosclerotic burden in a patient).^25^ The relation between diffuse coronary atherosclerosis and ischemia was emphasized earlier by De Bruyne et al.^26^

Our findings show that the plaque characteristics representing total atherosclerotic burden were higher in ischemic lesions, but only stenosis severity and lesion length were independent determinants for ischemia. Although promising, the exact cut-off values for new plaque parameters should be investigated more extensively in future studies.

## Limitations

As adenosine stress myocardial CT perfusion has only recently been implemented, the small sample size of this single center analysis is a limitation. With this first-pass contrast enhancement technique, acquisition timing is crucial to be able to detect attenuation differences between ischemic and normal myocardium. Furthermore, most patients only underwent adenosine stress myocardial CTP if obstructive CAD was suspected based on visual analysis, which could have introduced selection bias by creating a population with high CAD burden. Interpretation of adenosine stress myocardial CTP images is challenging, mainly due to the presence of motion and beam-hardening artifacts which can mimic and mask hypo-perfusion, potentially reducing diagnostic accuracy. Although studies of insufficient quality were excluded, image artifacts could have biased our results.

## New Knowledge Gained

This study explored the relationship between coronary CTA-derived plaque characteristics and ischemia by stress myocardial CTP. Quantitatively measured stenosis severity was moderately correlated with ischemia. Stenosis severity and lesion length were independent correlates, adjusted for mean plaque burden, plaque volume, maximal plaque thickness, and dense calcium volume.

## Conclusion

Increasing stenosis percentage by quantitative CTA is positively correlated to myocardial ischemia measured with adenosine stress myocardial CTP. However, stenosis percentage remains a moderate determinant for ischemia.

Coronary plaque volume, mean plaque burden, lesion length, maximal plaque thickness, and dense calcium volume were all significantly associated with myocardial ischemia, but only lesion length and stenosis severity were independent determinants.

## Abbreviations

APV: Aggregate plaque volume
CTA: Computed tomography angiography
CTP: Myocardial-computed tomography perfusion
FFR: Fractional flow reserve
ICA: Invasive coronary angiography
LAD: Left anterior descending coronary artery
LCX: Left circumflex coronary artery
MLA: Minimal lumen area
RCA: Right coronary artery
SPECT: Single-photon emission-computed tomography
